# Associations between venous thromboembolism onset, D-dimer, and soluble fibrin monomer complex after total knee arthroplasty

**DOI:** 10.1186/s13018-015-0315-4

**Published:** 2015-11-10

**Authors:** Genya Mitani, Tomonori Takagaki, Kosuke Hamahashi, Kenji Serigano, Yutaka Nakamura, Masato Sato, Joji Mochida

**Affiliations:** Department of Orthopaedic Surgery, Tokai University Oiso Hospital, Gakkyou 21-1, Oiso, Naka-gun, Kanagawa 259-0198 Japan; Department of Orthopaedic Surgery, Surgical Science, Tokai University School of Medicine, 143 Shimokasuya, Isehara, Kanagawa 259-1193 Japan; Department of Physical Recreation, School of Physical Education, Tokai University, 4-1-1 Kitakaname, Hiratsuka, Kanagawa 259-1292 Japan

**Keywords:** Minimally invasive total knee arthroplasty, Venous thromboembolism, Soluble fibrin monomer complex, D-dimer

## Abstract

**Background:**

Prevention and early detection of venous thromboembolism (VTE) is important after arthroplasty of the lower limb. The purpose of this study was to investigate the associations between VTE and hemostatic markers after minimally invasive total knee arthroplasty (MIS-TKA).

**Methods:**

We performed a retrospective study of 50 patients (55 knees) who underwent primary unilateral MIS-TKA with periodic determination of D-dimer and soluble fibrin monomer complex (SFMC) concentrations and with ultrasonography. The development of symptomatic and asymptomatic VTE, location of deep venous thrombosis (DVT; proximal or distal), changes in SFMC and D-dimer concentrations, and correlations between hemostatic markers and VTE onset were evaluated.

**Results:**

Twenty-six patients (47 %) had an asymptomatic distal DVT, but none had proximal DVT, pulmonary embolism, or symptomatic DVT. DVT was detected at postoperative day 1 (POD1) in 16 patients, POD3 in six, and POD5 in three (excluding detections of the same DVT in the same position on different days). DVT onset correlated significantly with SFMC concentration on POD1 and with D-dimer concentration on POD3. The D-dimer concentration did not differ significantly between patients who developed DVT (DVT+) and those who did not (DVT−) at each postoperative time. SFMC concentration differed between DVT+ and DVT− patients only on POD1. Analysis of each hemostatic marker classified as either within or outside the normal concentration range showed no significant correlations between D-dimer concentration and DVT onset at each period. There were significant correlations between SFMC concentrations and DVT onset on POD1 and POD3. There were also significant correlations between D-dimer positive (+) findings and/or SFMC+ findings and DVT onset on POD1 and POD3. D-dimer+ and/or SFMC+ findings had better specificity on POD1 and a positive predictive value on POD1 and POD3 compared with SFMC+ alone.

**Conclusions:**

SFMC concentration is an effective hemostatic marker for early detection of DVT. D-dimer concentration alone has limited value as a hemostatic marker for early detection of DVT. Measurement of both D-dimer and SFMC concentrations might be a more sensitive diagnostic tool than measuring SFMC concentration alone.

## Background

Reducing the rate of perioperative complications is crucial for performing safe surgery. One such complication is venous thromboembolism (VTE), which can have a fatal outcome. Surgeons are interested in ways of preventing VTE associated with arthroplasty of the lower limb. Early detection of asymptomatic deep vein thrombosis (DVT) is important because this is a major cause of pulmonary embolisms (PE) [1].

The use of DVT screening with imaging methods that involve radiation exposure has been reported, but these methods are invasive [2, 3]. This type of screening is also performed at fixed times after surgery. However, it should be performed only periodically in the early detection of indeterminate DVT. For consistent examination of DVT, the method needs to be less invasive and cause limited exposure of the patient to radiation; for this, measurement of hemostatic markers is considered to be a useful alternative. Boneu et al. recommend measurements of D-dimer concentration for monitoring or identifying the hypercoagulable state [4]. D-dimer is a small protein fragment that is present in the blood after a blood clot is degraded by fibrinolysis. It is so named because it contains two cross-linked D fragments of the fibrin protein [5]. Since its introduction in the 1990s, measurement of D-dimer concentration has become an important test in patients with a suspected thrombotic disorder. However, its usefulness for diagnosing VTE is controversial because of the high rate of false-positive results after invasive surgery and the difficulty in early detection. Chen et al. reported that the serum D-dimer concentration alone was not accurate enough to detect DVT after total knee arthroplasty (TKA) [6]. Schouten et al. reported that the use of age-adjusted cutoff values for D-dimer concentration substantially increased the specificity without modifying the sensitivity [7].

Soluble fibrin monomer complex (SFMC) has recently been identified as a quick and useful factor for detecting thrombophilia [8]. Although its measurement is associated with some false-positive results, SFMC concentration is detectable from an early phase of VTE [9], but its sensitivity is low when measured more than 3 days after the onset.

The purpose of this study was to determine whether there is an association between the development of VTE and hemostatic marker levels after minimally invasive TKA (MIS-TKA).

## Materials and methods

### Patients

This study complied with the Declaration of Helsinki (2013) and was approved by the Institutional Review Board for Clinical Research of Tokai University School of Medicine (ref. 15R-096). One hundred twenty-seven patients received a MIS-TKA for osteoarthritis or rheumatoid arthritis between January 2009 and June 2014. We performed a retrospective study of 50 of these patients (8 men; 42 women; 55 knees) who received periodic determination of D-dimer and SFMC concentrations and underwent ultrasonography (US) examinations. Their mean age was 70.9 ± 7.4 years. All patients were Asian. The exclusion criteria were a revision TKA, simultaneous bilateral TKA, a severely deformed knee needing augmentation implants, or contraindications for receiving an autologous blood donation.

### Surgical treatment and perioperative prophylaxes for VTE

We performed MIS-TKA for all patients at an accessible level while they were under general anesthesia. A tourniquet was not used for any patient. A slightly medial straight skin incision was made from the superior pole of the patella to the tibial tuberosity (range 8–10 cm long for individual cases). The mini-subvastus approach without patella eversion was used in all patients, and bone cutting was performed according to the MIS Quad-Sparing TKA technique [10].

In all cases, the prosthesis was a NexGen Complete Knee Solution Legacy Knee Posterior Stabilized (LPS) LPS-Flex Fixed Bearing Knee (Zimmer, Warsaw, IN, USA). In each case, the patella was resurfaced, and all components were fixed with cement. A total of 800 g of autologous blood was prepared before the operation, and this was transfused to each patient after surgery (400 g transfused twice). An epidural catheter was inserted during the operation and remained in place for 48 h after. Intermittent pneumatic compression was applied for the same period. The anticoagulant fondaparinux (Arixtra; GlaxoSmithKline plc, London, UK) was administered for 14 days (2.5 mg daily) from 24 h after removal of the epidural catheter. All patients received rehabilitation therapy sessions each lasting 60–120 min per day on 5–6 days per week throughout their hospitalization, and clinical symptoms were observed every day until discharge.

### Measurements of D-dimer and SFMC concentrations

The serum concentrations of D-dimer (CS-2100i; Sysmex, Hyogo, Japan) and SFMC (STACIA; LSI Medience, Tokyo, Japan) were measured to detect hypercoagulability using the latex agglutination method on the day before the operation and on postoperative days (PODs) 1, 3, 5, 7, 10, and 14.

### Ultrasonography

Bilateral US examination of the calf, popliteal fossa, and thigh was performed using a LOGIQ E9 instrument (GE Healthcare, Tokyo, Japan). The B mode scan with compression and the color Doppler method were performed using a linear probe with a frequency of 7.5 MHz. The criteria for the diagnosis of DVT were the presence of intraluminal thrombotic echogenicity and lack of venous compressibility. The color Doppler method was used for anatomical orientation and venous examination but not for the diagnosis of DVT. All sonographers were trained. DVT was classified as being in either a proximal (i.e., the popliteal vein or any one proximal to it) or a distal vein (any vein distal to the popliteal one).

### Evaluations

The rate of development of symptomatic and asymptomatic VTE, location of the DVT (proximal or distal), changes in the concentrations of SFMC and D-dimer, and correlations between each hemostatic marker and VTE onset were evaluated.

### Statistical analysis

Logistic regression analysis was used to examine the relationship between each marker and DVT onset on each day in the total group of patients and in patients grouped according to whether the marker concentration was within or below the normal range. The Mann–Whitney nonparametric *U* test was used to detect differences between markers in the patients with any DVT (DVT+) and without DVT (DVT−). The Tukey test was used to evaluate any changes in the concentrations of the two markers in relation to the onset of DVT. All tests were two-sided, and *p* < 0.05 was considered significant. All data processing and analysis were performed using SPSS statistical software (version 17.0; SPSS Inc., Chicago, IL, USA).

## Results

Twenty-six patients (47 %) had asymptomatic distal DVT, but none had proximal DVT, PE, or symptomatic DVT. DVT was detected in 16 patients (29 %) on POD1, six (11 %) on POD3, and three (5 %) on POD5 (excluding detections of the same DVT in the same position on different days). Asymptomatic distal DVT was treated with normal postoperative rehabilitation without bed rest or additional anticoagulant therapy, and no patient showed extension of the detected distal DVT to a proximal DVT.

D-dimer and SFMC concentrations were measured as possible hemostatic markers for detecting VTE. Initially, we focused on the values at each POD. The mean D-dimer concentrations for all patients were 2.4 ± 4.0 μg/mL before the operation, 12.6 ± 13.0 μg/mL on POD1, 8.7 ± 5.9 μg/mL on POD3, 11.3 ± 6.3 μg/mL on POD5, 11.6 ± 4.0 μg/mL on POD7, 10.3 ± 3.5 μg/mL on POD10, and 9.6 ± 4.9 μg/mL on POD14.

The mean SFMC concentrations for all patients in all cases were 7.3 ± 8.9 μg/mL before the operation, 25.4 ± 24.0 μg/mL on POD1, 16.3 ± 15.6 μg/mL on POD3, 10.3 ± 13.9 μg/mL on POD5, 6.9 ± 11.0 μg/mL on POD7, 8.3 ± 11.2 μg/mL on POD10, and 8.8 ± 15.4 μg/mL on POD14.

DVT onset correlated significantly with the SFMC concentration on POD1 (*p* = 0.0012) and with the D-dimer concentration on POD3 (*p* = 0.0428; Table 1).

**Table 1:** Correlations between DVT onset and D-dimer and SFMC concentrations throughout the course of the study

	*B*	*p*	Odds ratio
D-dimer POD1	1.05	0.126	2.87
D-dimer POD3*	1.13	0.043	3.10
D-dimer POD5	1.07	0.202	2.91
D-dimer POD7	1.09	0.254	2.96
D-dimer POD10	1.11	0.235	3.02
D-dimer POD14	0.98	0.774	2.67
SFMC POD1**	1.06	0.001	2.88
SFMC POD3	1.02	0.348	2.77
SFMC POD5	1.04	0.223	2.82
SFMC POD7	1.00	0.890	2.71
SFMC POD10	1.00	0.875	2.71
SFMC POD14	0.98	0.400	2.67

*B* unstandardized correlation coefficient

*p < 0.05; **p < 0.01

We next classified the patients according to whether DVT was detected (DVT+) or not (DVT−). The mean D-dimer concentrations in the DVT− group were 2.6 ± 4.9 μg/mL before the operation, 9.1 ± 7.3 μg/mL on POD1, 7.1 ± 3.9 μg/mL on POD3, 10.6 ± 5.1 μg/mL on POD5, 11.3 ± 3.8 μg/mL on POD7, 9.9 ± 3.7 μg/mL on POD10, and 10.4 ± 6.7 μg/mL on POD14.

**Fig. 1:**
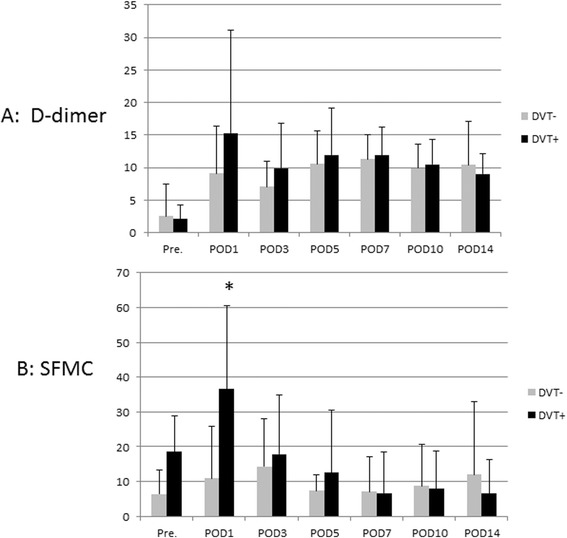
Comparison of D-dimer and SFMC concentrations between the DVT+ and DVT− groups. **a** D-dimer concentration did not differ significantly between the DVT+ and DVT− groups at any time. **b** SFMC concentration differed between the DVT+ and DVT− groups only on POD1 (*p < 0.001)

The mean D-dimer concentrations in the DVT+ group were 2.1 ± 2.1 μg/mL before the operation, 15.3 ± 15.8 μg/mL on POD1, 9.9 ± 6.8 μg/mL on POD3, 11.9 ± 7.1 μg/mL on POD5, 11.9 ± 4.3 μg/mL on POD7, 10.5 ± 3.9 μg/mL on POD10, and 9.0 ± 3.1 μg/mL on POD14. D-dimer concentrations did not differ significantly between the DVT+ and DVT− groups on any POD (Fig. 1a).

The mean SFMC concentrations in the DVT− group were 6.4 ± 6.9 μg/mL before the operation, 10.9 ± 14.9 μg/mL on POD1, 14.3 ± 13.8 μg/mL on POD3, 7.4 ± 4.7 μg/mL on POD5, 7.1 ± 10.2 μg/mL on POD7, 8.7 ± 12.1 μg/mL on POD10, and 12.0 ± 20.9 μg/mL on POD14.

The mean SFMC concentrations in the DVT+ group were 8.5 ± 10.5 μg/mL before the operation, 36.6 ± 23.9 μg/mL on POD1, 17.8 ± 16.9 μg/mL on POD3, 12.6 ± 17.8 μg/mL on POD5, 6.7 ± 11.8 μg/mL on POD7, 7.9 ± 10.8 μg/mL on POD10, and 6.5 ± 9.8 μg/mL on POD14. The SFMC concentration differed significantly between the DVT+ and DVT− groups only on POD1 (*p* < 0.001; Fig. 1b).

**Fig. 2:**
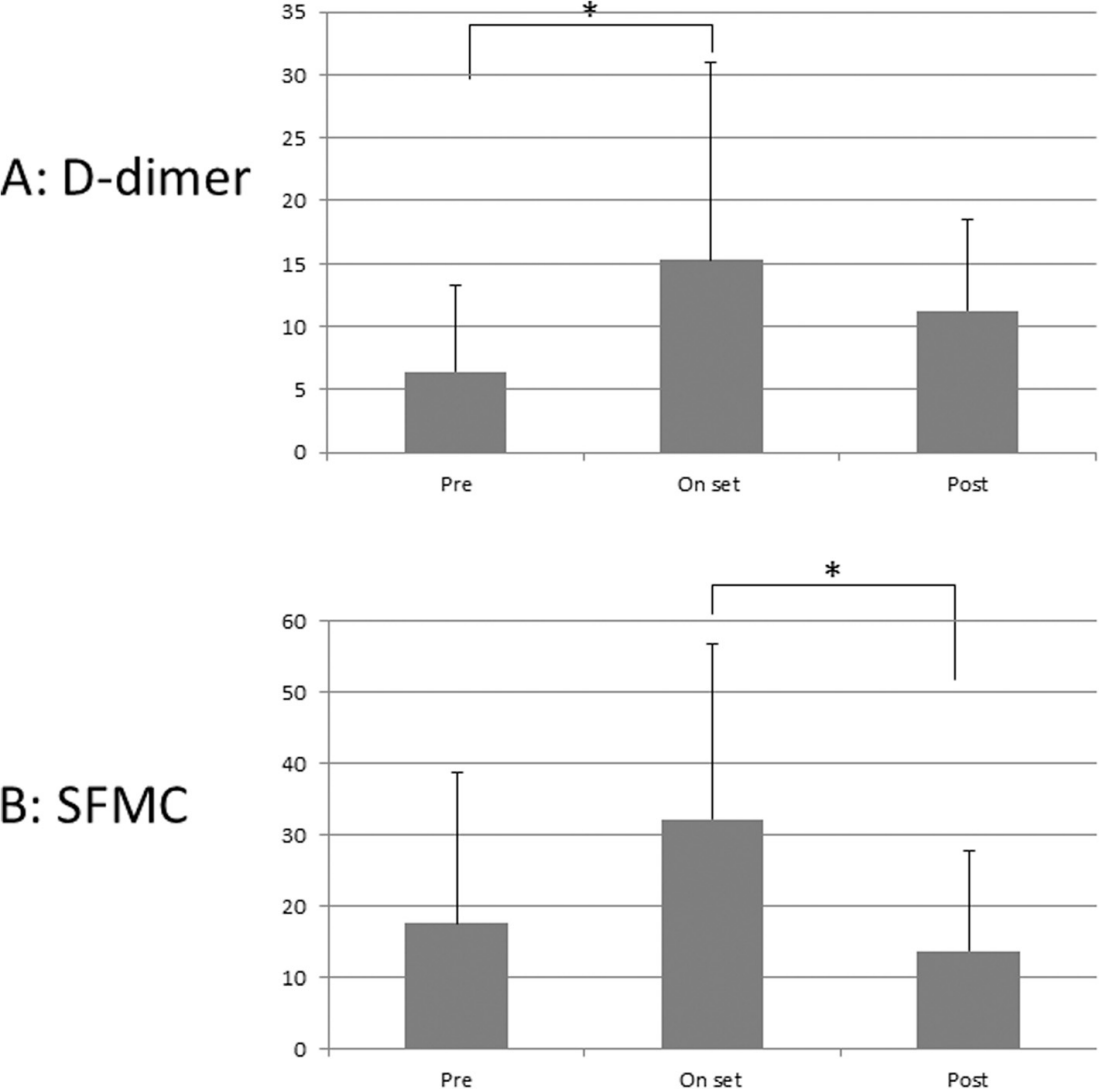
Changes in D-dimer and SFMC concentrations in relation to the day of DVT detection. **a** D-dimer concentration differed significantly between the pre and onset groups (*p < 0.01). **b** SFMC concentration differed significantly between the onset and post groups (*p < 0.01)

We evaluated the changes in the concentrations of the two markers in relation to the onset of DVT. In the analysis, the detection day is called “onset,” 2 days before the onset is called “pre,” and 2 days after the onset is called “post.” The mean D-dimer concentrations were pre, 6.5 ± 6.9 μg/mL; onset, 15.3 ± 15.7 μg/mL; and post, 11.3 ± 7.3 μg/mL. The mean SFMC concentrations were pre, 17.6 ± 21.2 μg/mL; onset, 32.2 ± 24.6 μg/mL; and post, 13.7 ± 14.1 μg/mL. D-dimer concentration differed significantly between the pre and onset sets (Fig. 2a). SFMC concentration differed significantly between the onset and post sets (Fig. 2b).

We next examined the efficacy of each hemostatic marker for DVT detection when classified as either within the normal level or below the normal level. The normal concentrations used were <5.4 μg/mL for D-dimer and <7.0 μg/mL for SFMC. We classified the patients according to whether their D-dimer and SFMC concentrations were within or outside the normal level, as D-dimer+, SFMC+, and D-dimer+ and/or SFMC+.

**Fig. 3:**
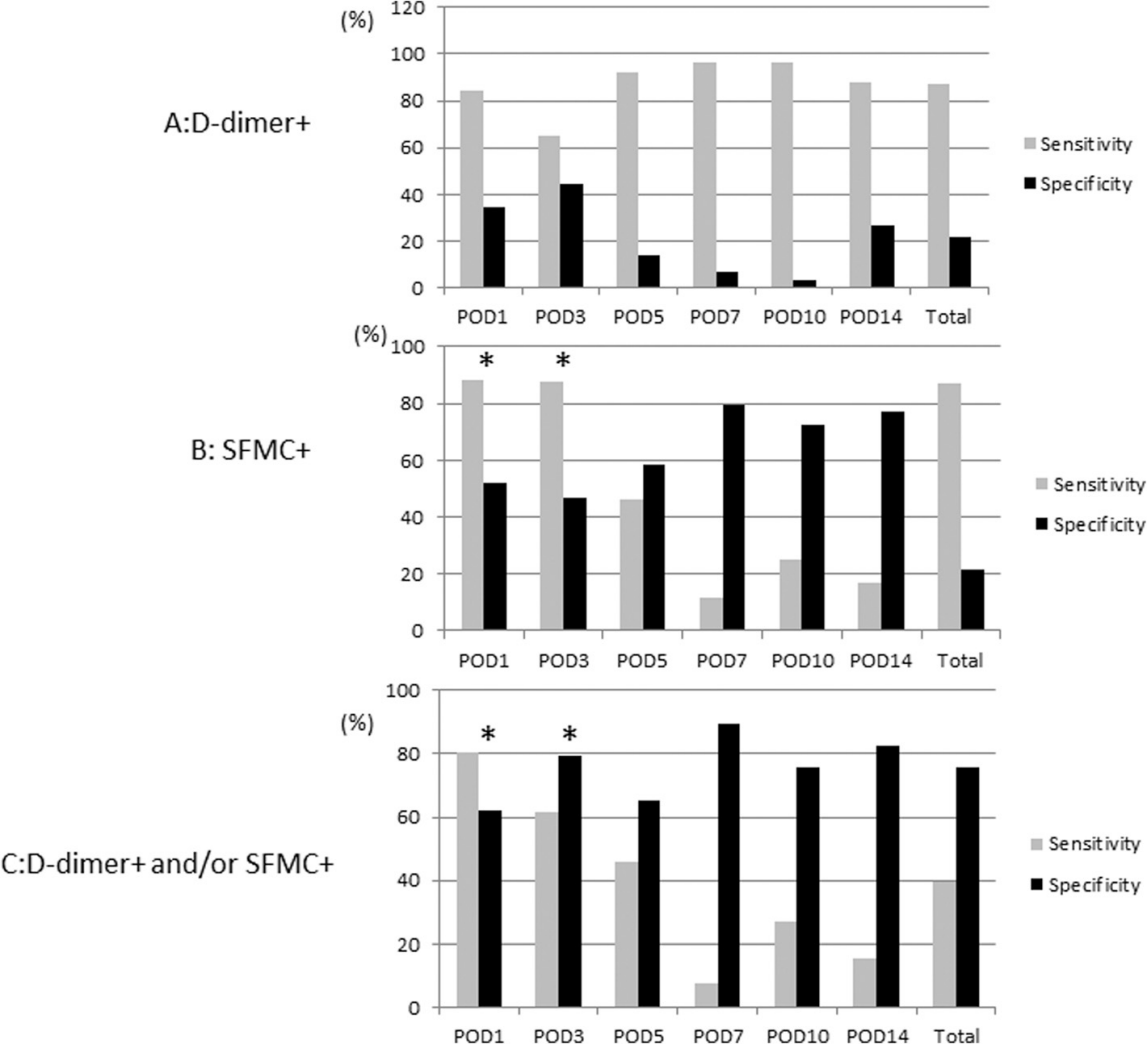
Sensitivity and specificity of two markers grouped as within/outside the normal range. **a** The D-dimer+ measure did not correlate significantly with DVT onset during any period. **b** The SFMC+ measure at POD1 and POD3 correlated significantly with DVT onset for sensitivity, specificity, and odds ratio (*p < 0.05). **c** The D-dimer and/or SFMC+ measures at POD1 and POD3 correlated significantly with DVT onset for sensitivity, specificity, and odds ratio; the SFMC+ only measure showed a similar pattern (*p < 0.05)

Table 2 shows the correlations between each marker and DVT onset for each day. There was no correlation between D-dimer+ and DVT onset in each period (Fig. 3a). SFMC+ on POD1 and POD3 correlated significantly with DVT onset (Fig. 3b). In addition, there was significant correlation between D-dimer+ and/or SFMC+ and DVT onset at POD1 and POD3; the same pattern was seen for SFMC+ only (Fig. 3c).

**Table 2:** Correlation between DVT onset and two markers grouped as within/outside the normal range

	*B*	*p*	Odds ratio	95 % CI
D-dimer+ POD1	1.06	0.189	2.89	0.77–10.74
D-dimer+ POD3	0.43	0.619	1.53	0.51–4.56
D-dimer+ POD5	0.65	0.77	1.92	0.32–11.47
D-dimer+ POD7	0.62	0.922	1.85	0.15–21.70
D-dimer+ POD10	−0.11	0.52	0.89	0.05–15.03
D-dimer+ POD14	0.99	0.322	2.70	0.61–11.93
D-dimer+ total	0.62	0.053	1.86	1.02–3.37
SFMC+ POD1*	2.11	0.003	8.21	2.01–33.52
SFMC+ POD3*	2.34	0.004	10.4	2.05–52.68
SFMC+ POD5	0.19	0.932	1.21	0.41–3.53
SFMC+ POD7	−0.69	0.581	0.50	0.11–2.24
SFMC+ POD10	−0.13	0.936	0.88	0.26–2.85
SFMC+ POD14	−0.40	0.831	0.67	0.16–2.72
SFMC+ total	0.62	0.053	1.86	1.02–3.37
D-dimer+ and/or SFMC+ POD1*	1.93	0.003	6.87	2.00–23.52
D-dimer+ and/or SFMC+ POD3*	1.81	0.004	6.13	1.85–20.29
D-dimer+ and/or SFMC+ POD5	0.49	0.544	1.63	0.54–4.82
D-dimer+ and/or SFMC+ POD7	−0.32	0.898	0.72	0.11–4.70
D-dimer+ and/or SFMC+ POD10	0.15	0.941	1.16	0.34–3.89
D-dimer+ and/or SFMC+ POD14	−0.13	0.857	0.87	0.20–3.67
D-dimer+ and/or SFMC+ total	0.73	0.003	2.07	1.29–3.32

*B* unstandardized coefficient, *CI* confidence interval

*p < 0.05

## Discussion

D-dimer and SFMC differ in their clinical significance. SFMC reflects the very early stage of a thrombotic event, whereas D-dimer reflects secondary fibrinolysis after clot formation [11, 12]. Ota et al. reported that measurement of the levels of both D-dimer and SFMC increased both sensitivity and specificity for diagnosing DVT or PE [13]. Ultrasonography (US) is a useful method that causes no radiation exposure, is minimally invasive, and is suitable for frequent follow-up of detected DVT even after TKA. Kraay et al. reported that US could be used to identify patients with asymptomatic proximal DVT [14]. Wada et al. reported on the effectiveness of hemostatic markers and US for screening for DVT [15].

Recent studies have reported the rates of VTE after TKA. Bauer et al. reported that VTE occurred after TKA in 12.5 % of patients [16]. Fuji et al. [17] reported a VTE rate of 16.2 % in patients given fondaparinux anticoagulation therapy. Chen et al. [6] reported a rate of 40 % in Asian patients not given chemical thromboprophylaxis, and Migita et al. [18] reported a rate of 24.3 % in patients with or without such treatment. The 47.3 % of patients who developed DVT in our study of Asian people was higher than the rate reported in other studies, especially because all patients received fondaparinux. However, symptomatic DVT, proximal DVT, and PE were not detected in our patients, and we performed more frequent screening than in other studies. We believe that the combination of periodic checks using both hemostatic markers and US allowed us to detect asymptomatic distal DVT, which might have been missed in other studies.

There is debate about whether anticoagulant therapy is necessary in the management of distal DVT [19]. Palareti et al. [20] and Hara et al. [21] reported that asymptomatic distal DVT does not need extra anticoagulant treatment because of the low risk of progression to proximal DVT or PE. In our study, patients with asymptomatic distal DVT were given normal rehabilitation without bed rest or extra anticoagulant therapy, but there were no cases of spreading of DVT to the proximal veins and/or onset of PE. Thus, we conclude that this management is acceptable for patients with asymptomatic distal DVT.

We found significant correlations between DVT onset and SFMC concentration on POD1 (*p* = 0.0012) and D-dimer concentration on POD3 (*p* = 0.0428). These results are consistent with a high onset rate of DVT on POD1 and a second peak for each marker, given that SFMC reflects the early stage of a thrombotic event and D-dimer reflects secondary fibrinolysis after clot formation.

Our evaluation of two markers in relation to the onset of DVT confirmed their predictive value, as reported previously [9, 13]. D-dimer concentration tended to remain high after the onset of DVT, whereas SFMC concentration tended to be high because of the hypercoagulable state before DVT onset but decreased quickly after DVT onset. Each of these features of the two markers should be considered when diagnosing DVT.

We compared the D-dimer and SFMC concentrations in patients classified according to whether they did or did not develop DVT (DVT+ and DVT− groups, respectively). D-dimer concentration did not differ significantly between groups on any day. SFMC concentration differed significantly between groups only on POD1. These results suggest that SFMC concentration might be valuable for the early detection of DVT, but the D-dimer concentration is not.

We also compared the D-dimer and SFMC concentrations in patients classified according to whether the marker was within the normal concentration range. In this analysis, the D-dimer+ result and DVT onset did not correlate significantly on any day. SFMC+ and DVT onset correlated significantly on POD1 and POD3. There were significant correlations between D-dimer+ and/or SFMC+ and DVT onset on POD1 and POD3.

Intriguingly, D-dimer+ and/or SFMC+ (sensitivity, 81 %; specificity, 62 %; positive predictive value, 65 %) had better specificity and positive predictive value compared with SFMC+ only (sensitivity, 88 %; specificity, 51 %; positive predictive value, 62 %) on POD1. D-dimer+ and/or SFMC+ had a better positive predictive value (72 %) compared with SFMC+ only (48 %) on POD3.

These results suggest that SFMC may be an effective hemostatic marker for the early detection of DVT because of its significant correlation with DVT onset because most DVTs were detected during this period. In particular, SFMC concentration on POD1 correlated strongly with the onset of DVT. Thus, the D-dimer concentration alone has limited value as a hemostatic marker for early detection of DVT, but measurement combining both D-dimer and SFMC concentrations might be a more sensitive diagnostic tool than measurement of SFMC concentration alone. Thus, measurement of both SFMC and D-dimer concentrations can be recommended for detecting VTE when the onset is unknown because the SFMC concentration tends to decrease about 2 days after the onset of VTE.

Overall, the results from our study of Asian patients after MIS-TKA suggest that there is a low risk of proximal DVT or PE in patients treated without a tourniquet or thromboprophylaxis with fondaparinux after removal of the epidural catheter. Therefore, periodic measurements of both SFMC and D-dimer concentrations might be a prudent way to monitor patients in such situations. We recommend periodic measurement of both SFMC and D-dimer concentrations in patients when there is suspicion of DVT or PE and after surgery for high-risk patients.

There were some limitations to this study. First, this was a retrospective study under only one condition and was not a randomized or controlled study. Second, the diagnosis of DVT by US was not corroborated by another method. Although all sonographers were trained adequately, we accept that the quality of the physician’s training might influence the accuracy of detecting a DVT. Third, hemostatic markers were measured every second day after the operation in this study, so it is possible that a DVT had developed on the day before that recorded as being the actual onset.

## Conclusions

SFMC might be an effective hemostatic marker for early detection of DVT. SFMC concentration on POD1 correlated strongly with the onset of DVT. D-dimer concentration alone had limited value as a hemostatic marker for the early detection of DVT. Measurement of D-dimer and SFMC concentrations combined might provide a more sensitive tool for diagnosing DVT than measurement of SFMC concentration alone.

## Abbreviations

DVT: deep vein thrombosis
MIS-TKA: minimally invasive total knee arthroplasty
PE: pulmonary embolisms
POD: postoperative day
SFMC: soluble fibrin monomer complex
US: ultrasonography
VTE: venous thromboembolism
